# Risk factors for new renal scarring in children with vesicoureteral reflux receiving continuous antibiotic prophylaxis

**DOI:** 10.1038/s41598-024-52161-w

**Published:** 2024-01-20

**Authors:** Dequan Su, Zhiqiang Zhuo, Jinqiang Zhang, Zhuqin Zhan, Honglin Huang

**Affiliations:** Six Ward, Xiamen Children’s Hospital/Children’s Hospital of Fudan University Xiamen Branch, Xiamen, 361006 Fujian China

**Keywords:** Medical research, Nephrology

## Abstract

To investigate the risk factors for new renal scarring (NRS) in children with vesicoureteral reflux (VUR) receiving continuous antibiotic prophylaxis (CAP). This was a single-center cohort study. The clinical data of 140 children with grade I–V VUR receiving CAP were analyzed. In this study, exposure variables were sex, younger age at the initial diagnosis of UTI ≤ 12 months, the occurrence of breakthrough urinary tract infection (BT-UTI), high-grade VUR, bilateral VUR, etiology, presence of renal scarring at the initial diagnosis and ultrasound abnormalities. The outcome was NRS. A total of 140 children were included in the risk factor analysis of NRS, 73 of whom experienced NRS, an incidence rate of 52.14%. Multivariate Cox regression suggested that the presence of renal function impairment after the initial diagnosis of UTI (OR 3.411, 95% CI 1.5751–6.646) and the occurrence of BT-UTI while receiving CAP (OR 1.995, 95% CI 1.089–2.958) were independent risk factors for NRS. Multivariate Cox regression showed that high-grade VUR had no significant effects on NRS (OR 0.940, 95% CI 0.462–1.912, P = 0.864). No significant difference was identified in multivariate Cox regression analysis in the IV–V group (vs I–III group) (OR 0.960, 95% CI 0.565–1.633, P = 0.960). Renal function impairment after the initial diagnosis of UTI and the occurrence of BT-UTI while receiving CAP were independent risk factors for NRS. Neither univariate analysis nor multivariate analysis found a correlation between VUR grade and NRS.

## Introduction

Vesicoureteral reflux (VUR) is a disease involving the reflux of urine from the bladder to the ureter or pelvis; VUR is one of the most common congenital kidney and urinary tract malformations in children, and children with VUR are at high risk for breakthrough urinary tract infection (BT-UTI), and 5‒10% of will experience decreased glomerular filtration rates. This leads to reflux nephropathy, which eventually leads to hypertension, albuminuria, chronic renal impairment, and even renal failure in some children^1–4^. Reflux nephropathy usually has no obvious clinical manifestations in the early stage, and its pathology is characterized by patchy interstitial scarring, tubular atrophy, and nephron atrophy. The mechanism of VUR-induced regurgitant nephropathy is not well understood. Repeated urinary tract infections and immune impairment are thought to contribute to regurgitant nephropathy^5,6^. Current therapeutic strategies for VUR are aimed at reducing the occurrence of new BT-UTIs and kidney function impairment^7,8^. Recurrent BT-UTI in VUR children is prone to new renal scarring, which can also affect their long-term prognosis, with the potential risk of developing hypertension, albuminuria, renal impairment, and even renal failure^6^. This study aimed to analyze the clinical data of children receiving CAP treatment for VUR in our hospital from 2016 to 2019, with the purpose of summarizing the risk factors for new kidney scarring in children with VUR.

## Patients and methods

**Figure 1:**
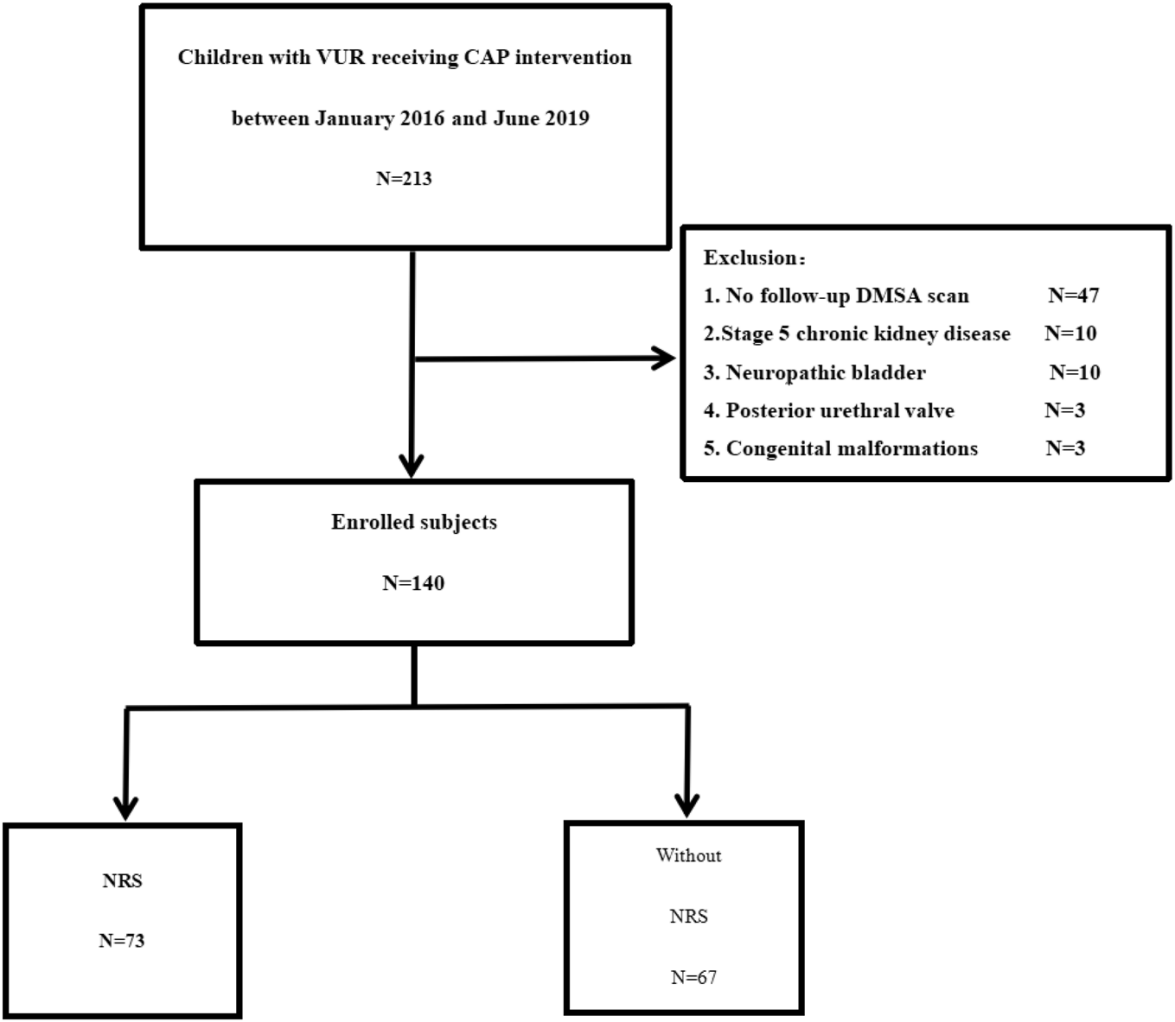
Selection and group information of the study subject.

This is a retrospective cohort study included 213 children with vesicoureteral reflux (VUR) receiving CAP study. Children seeking treatment due to febrile UTI at the outpatient clinic or inpatient ward of the Department of Nephrology, Children's Hospital of Xiamen, between January 2016 and June 2019 were enrolled in the study. These children were diagnosed with VUR by micturating cystourethrography (MCU) and received CAP intervention. They all receiving regular CAP and following-up by specialist every month through phone or outpatient service. The exclusion criteria were as follows: no follow-up DMSA scan, secondary VUR (neuropathic bladder, posterior urethral valve, congenital tethered cord syndrome), stage 5 chronic kidney disease, genital malformations (urethro-rectal fistula, recto-vesical fistula), and congenital immunodeficiency. A total of 140 children were included in the analysis of risk factors for NRS (Fig. 1). This cohort study was approved by the medical ethics committee of Children's Hospital of Xiamen. We confirmed that all methods were performed in accordance with the relevant guidelines and regulations. The diagnosis and grading were based on the VUR classification (grade I–V) developed by the International Reflux Study Committee^9^. The classification of bilateral VUR was determined according to the side with a higher reflux grade. After the diagnosis of VUR, all patients received CAP. CAP was given in a single oral dose per night (one-quarter to one-third of a daily dose). Antibiotics (nitrofurantoin enteric-coated tablets, sulfamethoxazole tablets, etc.) were selected according to the bacterial susceptibility and were orally administered. The treatment was individualized for each child, CAP is empirically recommended until age of toilet training. The diagnostic criteria of BT-UTI included: (1) body temperature higher than 38 ℃; cloudy, foul-smelling urine or symptoms such as urinary urgency, dysuria, and back pain; (2) abnormal midstream clean-catch urine laboratory values: leukocytes > 5/high-power field and positive urinary leukocyte esterase; (3) urine culture: bacterial growth in midstream urine > 10^5^ colony-forming units (CFU)/mL^5^. In our study, obtaining urine samples for non-toilet-trained children include clean catch, bag, in–out catheterisation.

### DMSA scan

**Figure 2:**
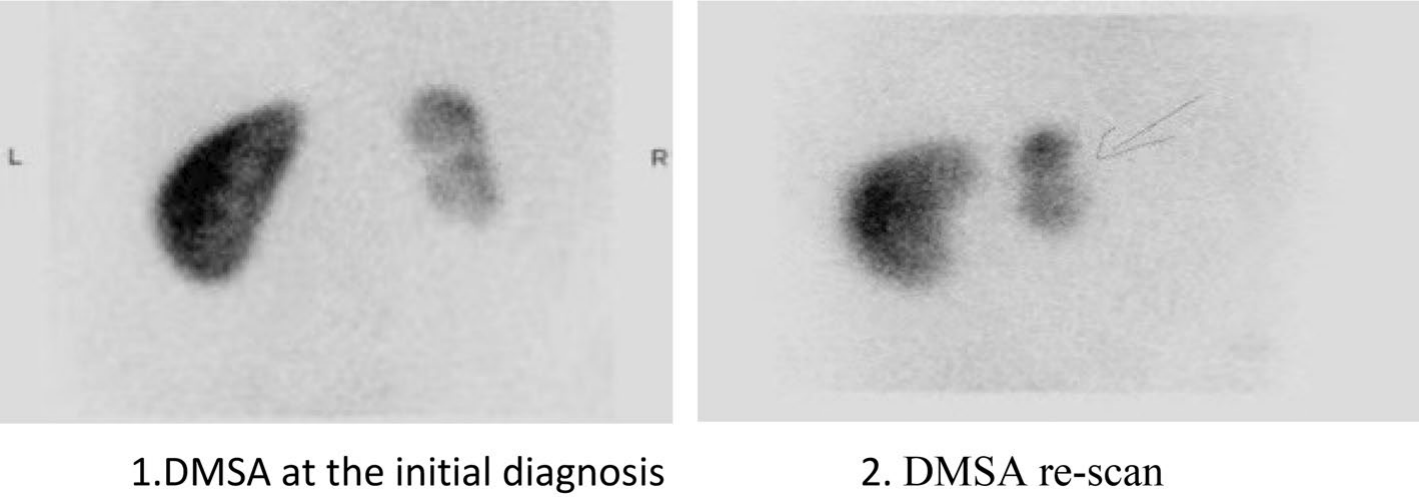
New renal scarring image example.

Kidney dimercaptosuccinic acid (DMSA) scan interpretation: 99mTc-DMSA renal static imaging was performed. After receiving an intravenous injection of 22.2–185.0 MBq imaging agent (1.85 MBq/kg body weight) for 3–4 h, children were placed in the supine position, and static imaging in the anteroposterior view was performed using a low-energy high-resolution parallel-hole collimator with a field of view that included both kidneys and the bladder, energy peak 140 keV, window width 20%, matrix 512 × 512, magnification 2.19, and radioactivity count 1.8 × 106. The 99mTc-DMSA renal imaging protocol and result assessment followed the clinical guideline of the Society of Nuclear Medicine and Molecular Imaging^10,11^. The time between index UTI and first DMSA was 1 (0.6) months. New renal scarring was assessed by DMSA performed minimally 12 months after the treatment of UTI, and median time was 16 (12,27)months. The interpretations of the DMSA was made by experienced nuclear medicine consultants and experienced radiologists. Normal imaging was considered to be a normal location, morphology, and contour of both kidneys and a uniform distribution of radioactivity in the renal parenchyma. The percentage of renal function on each side (differential renal function) was calculated by delineating the kidney regions of interest and background using computer, and a differential renal function greater than 10% was considered renal impairment. One or more focal radioactivity reductions or defects in the renal cortex, with contraction or volume reduction of the affected cortex, or wedge-shaped defects denoted renal scarring^11^. NRS was defined as the occurrence of new renal scarring or aggravation of existing renal scarring on a DMSA re-scan while receiving CAP^11^ (Fig. 2).

### Study design

The VUR children were divided into an NRS group and an NRS-free group based on the presence or absence of renal scarring observed during the follow-up DMSA scan. The risk factors that were analyzed were were sex, age, pathogens (*Escherichia coli* and non-*E. coli*) at the initial diagnosis of UTI, VUR grade, unilateral or bilateral VUR, presence or absence of renal scarring on a DMSA scan after the initial diagnosis of UTI, presence or absence of renal function impairment diagnosed by DMSA after the initial diagnosis of UTI, and ultrasound abnormalities.

### Statistical analysis

Measurement data are expressed as means ± standard deviation, and count data are presented as percentages (%). Univariate analysis was performed using the Kaplan–Meier analysis test for comparisons between groups. Cox regression was used to evaluate individual variables as independent risk factors for NRS. P < 0.05 was considered statistically significant. Data analysis was performed with SPSS 22.0 software.

### Ethics approval and consent to participate

The studies involving human participants were reviewed and approved by Ethical committee of Children Hospital of Xiamen (XMSETYY-2020-123). Written informed consent to participate in this study was provided by the participants’ legal guardian/next of kin.

## Results

### General information

A total of 140 children were included in this study, including 72 males and 68 females, with a median age of 5 months (range from 1 to 56 months). The median follow-up time was 18.3 months. None of the male patients had been circumcised. All VUR children received MCU after being diagnosed with febrile UTI. Of these patients, 70 had unilateral VUR, 70 had bilateral VUR, and VUR was present in a total of 210 ureters. There were 8 children with grade I VUR (5.71%), 14 with grade II (10%), 59 with grade III (42.14.%), 39 with grade IV (27.86%), and 20 with grade V (14.29%) ( Table 1).

**Table 1:** General information.

Characteristic	Number (%)
Total	140
Sex	
Male	70 (54.19)
Female	82 (45.82)
Age of initial urinary tract infection	
0 m < age≦12 m	91 (65)
≥ 12 m	49 (35)
VUR Grade	
I	8 (5.71)
II	14 (10)
III	59 (42.14)
IV	39 (27.86)
V	20 (14.29)
Unilateral	70 (50)
Bilateral	70 (50)
Number of UTIs before VCUG	
1	103 (73.57)
2	26 (18.57)
3	11 (7.86)

*VUR* vesicoureteral reflux, *VCUG* Voiding CystoUrethroGram.

### Baseline demographic and clinical characteristics

The 140 children who underwent DMSA re-scanning were divided into an NRS group and a non-NRS group for risk factor analysis. Chi-squared tests were used to compare the normal distribution between groups. The NRS group was found the presence of renal function improvement after the initial diagnosis of UTI and the presence of renal scaling at the initial diagnosis, with statistically significant differences between the groups (P < 0.05); There was no statistically significant difference between the other groups (P > 0.05) (Table 2).

**Table 2:** Baseline demographic and clinical characteristics.

Characteristic	NRC (n = 73)	Without NRC (N = 67)	P
Median, mo	4	5	
25th and 75th quartile, mo	4–13	3–13.5	
Age group			0.366
0–12mo	50 (35.71)	41 (29.29)	
> 12mo	23 (16.43)	26 (18.57)	
Sex			0.242
Male	41 (29.29)	31 (22.14)	
Female	32 (22.86)	36 (25.71)	
VUR grade			0.827
I–II	11 (7.85)	11 (7.85)	
III–V	62 (44.30)	56 (40.00)	
VUR grade			0.073
I–III	37 (26.42)	44 (32.43)	
IV–V	36 (25.71)	23 (16.43)	
VUR			0.398
Bilateral	39 (27.86)	31 (22.14)	
Unilateral	34 (24.29)	36 (25.71)	
Aetiology			0.448
*E. coli*	16 (21.62)	15 (20.27)	
Others	26 (35.14)	17 (22.97)	
DMSA at the initial diagnosis			0.000
Renal scarring	30 (21.43)	9 (6.43)	
No renal scarring	43 (30.71)	58 (41.43)	
DMSA at the initial diagnosis			0.000
Differential renal l function > 10%	61 (43.57)	25 (17.86)	
Differential renal function ≤ 10%	12 (8.57)	42 (30.00)	
Ultrasound abnormalities			0.647
Hydronephrosisg	23 (16.79)	19 (13.87)	
Ureter duplication	48 (35.04)	47 (34.30)	

*BT-UTI* breakthrough urinary tract infection, *NRC* new renal scarring, *VUR* vesicoureteral reflux, *E. coli* *Escherichia coli* Castellani, *DMSA* dimercaptosuccinic acid.

### Risk factors for NRS

Among the 140 children who received CAP, 73 experienced NRS, for an incidence rate of 52.14%. 53 children were detected one new scar, 15 were detected two new scars and 6 were detected three or more new scars. Univariate analysis showed that younger age at the initial diagnosis of UTI (≤ 12 months), the presence of renal function impairment after the initial diagnosis of UTI, BT-UTI and the presence of renal scarring at the initial diagnosis were correlated with NRS (P < 0.05). Multivariate Cox regression included the correlated factors with P < 0.05 in the univariate analysis and VUR grade. The results suggested that the presence of renal function impairment after the initial diagnosis of UTI (OR 3.411, 95% CI 1.751–6.646, P = 0.001) and the presence of BT-UTI while receiving CAP were independent risk factors for NRS (OR 1.995, 95% CI 1.089–2.958, P = 0.000). The 140 children were divided into a high VUR-grade group (III–V) and a low VUR-grade group (I–II).

Univariate analysis showed that high-grade VUR had no significant effects on NRS (P = 0.032). Multivariate Cox regression was also conducted, and no significant effect was identified (OR 0.940, 95% CI 0.462–1.912, P = 0.864). When the VUR children were divided into grade IV–V and grade I–III VUR groups, univariate analysis showed that children in the IV–V group also showed no significant differences in terms of NRS (P = 0.571). No significant difference was identified in multivariate Cox regression analysis (OR 0.960, 95% CI 0.565–1.633, P = 0.960) (Table 3).

**Table 3:** Risk factors for new renal scar formation in children with VUR during CAP intervention.

	Univariate Kaplan–Meier analysis			Multivariate Cox regression analysis		
	NRC (n = 73)	Without NRC (n = 67)	P	OR	95% CI	P
BT-UTI			0.001	1.995	1.089–2.958	0.000
Yes	45 (32.14)	13 (9.29)				
No	28 (20.00)	54 (38.57)				
VUR grade			0.891	0.940	0.462–1.912	0.864
I–II	11 (7.85)	11 (7.85)				
III–V	62 (44.30)	56 (40.00)				
VUR grade			0.571	0.960	0.565–1.633	0.960
I–III	37 (26.42)	44 (32.43)				
IV–V	36 (25.71)	23 (16.43)				
DMSA at the initial diagnosis			0.000	3.411	1.751–6.646	0.001
Differential renal function > 10%	61 (43.57)	25 (17.86)				
Differential renal function ≤ 10%	12 (8.57)	42 (30.00)				
DMSA at the initial diagnosis			0.003	1.255	0.754–2.090	0.383
Renal scarring	30 (21.43)	9 (6.43)				
No renal scarring	43 (30.71)	58 (41.43)				

*NRC* new renal scarring, *VUR* vesicoureteral reflux, *CAP* continuous antibiotic prophylaxis, *BT-UTI* breakthrough urinary tract infection, *E. coli* *Escherichia coli* Castellani, *DMSA* dimercaptosuccinic acid.

## Discussion

In this study, the clinical data of 140 children with grade I–V VUR receiving CAP at the Department of Nephrology, Children's Hospital of Xiamen were analyzed. Children were grouped based on the presence or absence of NRS while receiving CAP. Children with renal function impairment on a DMSA scan after the initial diagnosis of UTI and who had BT-UTI while receiving CAP were more prone to NRS.

In this study, the time between index UTI and first DMSA was 1(0.6) months. Loukogeorgakis et al.^12^ performed a retrospective analysis on 61 VUR children found that renal scarring is the most significant risk factor for breakthrough UTI in primary VUR patients and could be used to determine those at risk of symptomatic VUR persistence. Harper et al. also found that abnormal post-UTI DMSA scan is associated with a higher risk of recurrence of UTI at 24 months^13^. So a DMSA scan for VUR children after the first urinary tract infection is necessary.

In this study, children with VUR receiving regular CAP and specialist follow-up still had renal scarring. 140 children underwent a follow-up DMSA scan, and 73 children had NRS, including 41 males and 32 females, an incidence of 52.14%. Even when active clinical intervention is given to children with VUR, 30–50% of children still experience renal scarring, which is consistent with the findings^14,15^. In some recent randomized studies, the number of cases reported with renal scarring in children with VUR correlated with patient sex. Studies with more males reported significantly more cases of renal scarring, which is more likely to be congenital than acquired RN^16,17^. For girls, the risk of BT-UTI remains higher overall and the new scars were acquired and found to be related to severe inflammatory processes, while in boys the renal damage was often congenital^18^.

Univariate and multivariate analyses showed that in VUR children received CAP, the recurrence of BT-UTI was independent risk factor for NRS. Sevgi et al.^19^ performed a retrospective analysis on 90 VUR children receiving CAP and found that BT-UTI was a risk factor for renal scarring. Previous studies have suggested that renal parenchymal infection is a prerequisite for renal scarring^20,21^. This study showed that the rate of renal scarring in children with BT-UTI was 1.995 times that in children without BT-UTI recurrence, indicating that renal parenchymal infection is an important condition for renal scarring. But Morello et al.^22^ performed a investigator-initiated, randomized, open-label trial analysis on 292 grade IV or V VUR children receiving CAP and found that the number of new lesions was independent of the occurrence of UTIs during the trial period. They also founded that new defects were identified in 27 of 144 participants (18.8%) who had no UTI and in 11 of 57 (19%) who had at least one UTI (rate ratio, 0.97; 95% CI 0.52 to 1.83)^22^. Some studies have suggested that VUR children older than 2 years at the initial diagnosis of UTI have a greater likelihood of renal scarring, which may be related to the lack of timely treatment due to their late diagnosis of UTI^23,24^. In the RIVUR study, children with a second UTI had significantly more renal scars when compared with those with a single UTI^16^. A post hoc analysis of the RIVUR and CUTIE (Careful Urinary Tract Infection Evaluation) studies revealed that the odds of renal scarring after a second febrile UTI were 11.8 times higher and after 3 or more febrile UTIs were 13.7 times greater than after a single febrile infection^25^.

Previous studies have found that in children with VUR who use CAP intervention, high-level VUR, especially IV-V grade VUR, is an independent risk factor for NRS^26^. Mattoo et al.^5^ found in a prospective randomized controlled clinical study (RIVUR) that children with IV-V grade VUR were more likely to develop BT-UTI and renal scars, 24.2 and 1.88 times higher than those with I–III grade VUR, respectively. Sitarah Mathias et al.^27^ also found that patients with high-grade VUR patients were more likely than those with low-grade VUR patients to have renal scarring (75% vs. 49%, p < 0.01), low eGFR (23% vs. 13%, p Z 0.04) and significant hypertension (26% vs. 13%, p Z 0.02). In our study, neither the univariate nor multivariate analysis found a correlation between VUR grade and NRS. This result may be related to the high proportion of high-grade VUR children included in this cohort (84.28% of III–V grade VUR children and 42.14% of IV-V grade VUR children).

This study has some limitations. First, the study was a single-center, small cohort study. Second, although the children were regularly followed up in our hospital, there was no quantitative standard for CAP compliance. In addition, there were some data defects during data acquisition, such as the lack of etiology during the initial diagnosis of UTI. These factors may have led to biased results. Multicenter, prospective clinical studies are needed to confirm the reliability of our results.

## Conclusion

Renal function impairment after the initial diagnosis of UTI and the occurrence of BT-UTI while receiving CAP were independent risk factors for NRS. All of these factors require attention in clinical diagnosis and treatment.

## Data Availability

All data generated or analysed during this study are included in this published article.
